# Spatial distribution and associated factors of community based health insurance coverage in Ethiopia: further analysis of Ethiopian demography and health survey, 2019

**DOI:** 10.1186/s12889-022-13950-y

**Published:** 2022-08-10

**Authors:** Bewuketu Terefe, Tewodros Getaneh Alemu, Masresha Asmare Techane, Chalachew Adugna Wubneh, Nega Tezera Assimamaw, Getaneh Mulualem Belay, Tadesse Tarik Tamir, Addis Bilal Muhye, Destaye Guadie Kassie, Amare Wondim, Bethelihem Tigabu Tarekegn, Mohammed Seid Ali, Beletech Fentie, Almaz Tefera Gonete, Berhan Tekeba, Selam Fisiha Kassa, Bogale Kassahun Desta, Amare Demsie Ayele, Melkamu Tilahun Dessie, Kendalem Asmare Atalell

**Affiliations:** 1 Department of Community Health Nursing, School of Nursing, College of Medicine and Health Sciences, University of Gondar, Gondar, Ethiopia; 2 Department of Pediatrics and Child Health Nursing, School of Nursing, College of Medicine and Health Sciences, University of Gondar, Gondar, Ethiopia

**Keywords:** Community based health insurance coverage, Universal health coverage, Spatial distribution, EDHS 2019, Ethiopia

## Abstract

**Background:**

Community-Based Health Insurance is an emerging concept for providing financial protection against the cost of illness and improving access to quality health services for low-income households excluded from formal insurance and taken as a soft option by many countries. Therefore, exploring the spatial distribution of health insurance is crucial to prioritizing and designing targeted intervention policies in the country.

**Methods:**

A total of 8,663 households aged 15–95 years old were included in this study. The Bernoulli model was used by applying Kulldorff methods using the SaTScan software to analyze the purely spatial clusters of community based health insurance. ArcGIS version 10.3 was used to visualize the distribution of community-based health insurance coverage across the country. Mixed-effect logistic regression analysis was also used to identify predictors of community-based health insurance coverage.

**Results:**

Community based health insurance coverage among households had spatial variations across the country by regions (Moran’s I: 0.252, *p* < 0.0001). Community based health insurance in Amhara (*p* < 0.0001) and Tigray (*p* < 0.0001) regions clustered spatially. Age from 15–29 and 30–39 years (Adjusted Odds Ratio 0.46(AOR = 0.46, CI: 0.36,0.60) and 0.77(AOR = 0.77, CI: 0.63,0.96), primary education level 1.57(AOR = 1.57, CI: 1.15,2.15), wealth index of middle and richer (1.71(AOR = 1.71, CI: 1.30,2.24) and 1.79(AOR = 1.79, CI: 1.34,2.41), family size > 5, 0.82(AOR = 0.82, CI: 0.69,0.96),respectively and regions Afar, Oromia, Somali, Benishangul Gumuz, SNNPR, Gambella, Harari, Addis Ababa and Dire Dawa was 0.002(AOR = 0.002, CI: 0.006,0.04), 0.11(AOR = 0.11, CI: 0.06,0.21) 0.02(AOR = 0.02, CI: 0.007,0.04), 0.04(AOR = 0.04, CI: 0.02,0.08), 0.09(AOR = 0.09, CI: 0.05,0.18),0.004(AOR = 0.004,CI:0.02,0.08),0.06(AOR = 0.06,CI:0.03,0.14), 0.07(AOR = 0.07, CI: 0.03,0.16) and 0.03(AOR = 0.03, CI: 0.02,0.07) times less likely utilize community based health insurance than the Amhara region respectively in Ethiopia.

**Conclusion:**

Community based health insurance coverage among households in Ethiopia was found very low still. The government needs to develop consistent financial and technical support and create awareness for regions with lower health insurance coverage.

## Introduction

Community-Based Health Insurance (CBHI) is an emerging concept for providing financial protection against the cost of illness and improving access to quality health services for low-income households excluded from formal insurance [1]. CBHI is a policy considered a number one soft option by many countries and World Health Organization (WHO) to increase global health coverage. It is a guide for anyone who has a medical condition or wants to know more about the health check-up, as it will cover the total cost of the treatment in advance, even if they do not have many at the time of illness. As a result, people will be saved from the economic and social crisis that may ensue [2, 3]. This will make it easier and faster for countries to get out period, according to the World Bank [4]. Ethiopia has been making remarkable maternal and child health changes over the past decades. For example, the death toll among children under five has dropped from 47 to 25%, nevertheless, this is much less than expected by WHO [5]. In 2011, Ethiopia launched the community-based health insurance scheme initiative to extend health coverage to all [6].

In developing countries, access to high-quality, consumer-friendly healthcare services is at an all-time low. The challenge is even worse in Africa, especially sub-Saharan nations [7]. Countless people in the world do not have a chance to access primary medical care, and as a result, many of them die. Even in low- and middle-income countries alone, more than 150 million people suffer from health-related illnesses expenditure. More than two-thirds of these people suffer from chronic poverty and related problems [8, 9]. However, having a healthy population is the key to a country’s economic and social development [10, 11]. A study done in Ghana, Nigeria, Kenya and Tanzania showed that health insurance coverage remains low and needs special attention to enhance it. Age, level of education, residency area, income, and occupation were factors that made a difference in health insurance coverage [12]. In the same way, in Ethiopia, a study stated that health insurance coverage was around 39.89%; of these, more than 95% used their insurance. Trigger factors to join health insurance were the presence of illness in family members, education level, and low income [13].

According to a study conducted in Mekelle city, Ethiopia, it is commendable that Ethiopia has started providing community-based health insurance services because the cost of treatments is much higher than the income of the majority of the population. However, even though we benefit from the service, it does not have the necessary medicines and medical equipment’s, so it is purchased from private institutions. This has undermined public confidence in community-based health insurance [14]. A longitudinal study in Ethiopia revealed that the dropout rate after enrollment of CBHI is high across regions with a total present of 18% with a contract renewal rate of 82%, which can be considered a hindering factor. Lack of awareness was one factor of it [15].

Over the past several years, the healthcare system in Ethiopia is still a significant concern and requires much effort. For instance, the 2016 Ethiopia demographic and health survey data show that more than 90% of men and women did not have any health insurance. As a result, less than 2% of people receive health insurance from public and private health facilities [16, 17]. To address this crisis, the Ethiopian government has launched a national health sectors transformation plan for 2016–2020 and is working harder than ever by increasing healthcare financing, quality, equity, human resources and strategies that enable universal health coverage [18].

Different studies have been done in Ethiopia regarding willingness to pay to a CBHI. A study was done by Debre Markos town and Wolayita Sodo revealed that 69.8% and 71.3% among civil servants and teachers, respectively [19, 20]; however, in Addis Ababa, merely 17% have the willingness to pay [21]. This makes slow the succession path of universal health coverage plans. In Ethiopia, apart from health professionals, there is little public knowledge and attitudes of CBHI [22]. The enrollment to CBHI is influenced by many factors, like education level, awareness level, affordability of premium, perceived health status, perceived quality of treatment in public health facilities [22, 23].

A 20-year health sector development strategy guides the Federal Democratic Republic of Ethiopia's health system, which is implemented through a succession of five-year health sector development initiatives (HSDP). The fourth health sector development plan is now being implemented in the country (HSDP IV). The HSDP IV has implemented a three-tiered health-delivery system. Primary healthcare units (health posts, 3,000–5000 people, and health centres, 40,000 in urban, and 15,000–25,000 in rural areas) and primary hospitals (60,000–100,000 people in rural areas) provide primary level services; general hospitals provide secondary level services to 1–1.5 million people, and specialized teaching hospitals provide tertiary services to 3.5–5 million people [24].

CBHI is spatially varied, according to the Ethiopian Demographic and Health Survey 2019. Unfortunately, no spatial analysis has been undertaken among Ethiopian families to detect hotspots (poor health insurance coverage). As a result, we used the EDHS 2019 to evaluate the geographic variation in health insurance coverage and associated characteristics among Ethiopian households. It is critical to prioritize and plan targeted intervention programs to address universal health coverage through medical insurance by identifying geographic distributions of health insurance and the impact of risk factors on health insurance coverage by area. Furthermore, understanding the spatial variance of health insurance coverage is critical for creating community-based actions in recognized hotspot areas (poor health insurance coverage) and effectively deploying limited resources.

## Methods

### Study design, and setting

A community-based cross-sectional study was conducted in Ethiopia from March 21 to June 28, 2019. Ethiopia is a country in the Horn of Africa found in East Africa (3° -14° N and 33^0^ – 48° E) with nine regional states (Afar, Amhara, Benishangul-Gumuz, Gambella, Harari, Oromia, Somali, Southern Nations, Nationalities, and People’s Region (SNNP) and Tigray) and two city administrations (Addis Ababa and Dire Dawa). It has 68 zones, 817 districts, and 16,253 kebeles (lowest administrative units of a country). It has a population of over 110 million. Of which, 39.81% of the population are less than 14 years with a 1:1 sex ratio of the general population. The country also has a death rate of 5.8/1000, 22.2% of urbanization, with a very high degree of major infectious diseases [25, 26]. The present study used the recent Ethiopian Mini-Demographic and Health Survey 2019 (EDHS 2019) to determine the spatial distribution and determinants of community-based health insurance utilization in Ethiopia Ethiopian. Demographic Health Survey (EDHS) provides population and health indicators at the national and regional levels.

### Study population and sampling procedure

The 2019 EDHS sample was stratified and selected in two stages. Each region was stratified into urban and rural areas, yielding 21 sampling strata. Samples of Enumeration Areas (EAs) were selected independently in each stratum in two stages. Implicit stratification and proportional allocation were achieved at each lower administrative level by sorting the sampling frame within each sampling stratum before sample selection, according to administrative units in different levels, and using a probability proportional to size selection at the first stage of sampling [27].

To ensure survey precision is comparable across regions, the sample allocation has been done through an equal allocation where 25 EAs are selected from eight regions. However, the three more significant regions—Amhara, Oromia, and SNNP—35 EAs for each were selected [27].

In the first stage, a total of 305 EAs (93 in urban areas and 212 in rural areas) were selected with probability proportional to EA size (based on the 2019 population and housing census frame) and with independent selection in each sampling stratum. A household listing operation was carried out in all selected EAs from January through April 2019. The resulting lists of households served as a sampling frame for selecting households in the second stage. Some of the selected EAs for the 2019 Ethiopian Demographic and Health Survey (EDHS) were large, with more than 300 households. To minimize the task of household listing, each large EA selected for the 2019 EMDHS was segmented. Only one segment was selected for the survey, with probability proportional to the segment size. Household listing was conducted only in the selected segment; that is, a 2019 EMDHS cluster is either an EA or a segment of an EA. In the second stage of selection, a fixed number of 30 households per cluster were selected with an equal probability of systematic selection from the newly created household listing [27, 28].

### Data collection procedure and variables

The study was conducted based on EDHSs data by accessing the DHS program official database www. measuredhs.com after permission was granted through an online request by explaining the study's objective. The outcome variable with significant predictors was extracted from Ethiopia Demographic and Health Surveys household data set. Data were extracted using STATA version 14.0. Households were interviewed using questionnaires, based on the DHS Program’s standard questionnaires were adapted to reflect the population and health issues relevant to Ethiopia and several data from households were obtained. Socio-economic and demographic information was also collected from women and households. Health insurance coverage among households of all age was used as a dependent variable. The independent variables were age, level of education, residence, wealth status, occupation, family size and exposure to media, sex and region [4, 12, 29].

### Data processing and analysis

Descriptive and summary statistics were done using STATA version 14 software. The analysis was done using STATA 14, ArcGIS 10.3 and Sat Scan 9.6 software. The data were weighted using sampling weight, primary sampling unit, and strata before any statistical analysis to restore the survey's representativeness and tell the STATA to take into account the sampling design when calculating standard errors to get reliable statistical estimates.

In EDHS data, households within a cluster may be more similar than households in the rest of the country. This violates the assumption of independence of observations and equal variance across clusters. This implies that the need to consider the between-cluster variability using advanced models. Since the response variable was dichotomous, logistic regression and Generalized Linear Mixed Model (GLMM) were fitted. Model comparison was made based on Deviance Information Criteria (DIC). A mixed-effect model with the lowest DIC was chosen. Furthermore, the ICC value was 0.54, which informed us to choose GLMM over the basic model [30–32].

Variables with ≤ 0.2 p-values in the bi-variable analysis were fitted in the multivariable model to measure the effect of each variable after adjusting for the effect of other variables. Adjusted Odds Ratio (AOR) with a 95% Confidence Interval (CI) and p-value < 0.05 in the multivariable model were declared as determinant factors of community-based health insurance. Multi-collinearity was also checked using a variance inflation factor (VIF).

### Spatial scan statistical analysis

Spatial scan statistical analysis was employed to identify the geographical locations of statistically significant spatial clusters of community-based health insurance coverage among household people aged 15–95 years using Kuldorff’s Sat Scan version 9.6 software. Spatial scan statistics used a scanning window that moves across the study area. Scan statistics did scan gradually across the space to identify the number of observed and expected observations inside the window at each location. The scanning window with the maximum likelihood was the most likely high performing cluster, and a p-value was assigned to this cluster [33]. The maximum cluster size was set at 50% of the population at risk. Households without CBHI coverage were taken as controls, and those covered with CBHI were taken as cases represented by a 0/1 variable to fit the Bernoulli model. The number of cases in each location had Bernoulli distribution, and the model required data with or without health insurance coverage. A Likelihood ratio test statistic was used to determine whether the number of observed insured cases within the potential cluster was significantly higher than expected or not. Primary and secondary clusters were identified using p values and likelihood ratio tests based on the 999 Monte Carlo replications [33].

## Results

### Sociodemographic characteristics of the study population

A total of 8,663 household members were interviewed. The 5,999 (69.25%) of the respondents were rural residents, and the 6,751 and 2,189 (77.93% and 25.27%) of the study participant age were male and classified from 30–39 years respectively. Regarding the level of education, 4,120 (47.56%) of them were have no formal school. The household wealth of 24.44% of the study subjects was in the richest wealth quintiles, 17.30% were in the poorest wealth quintiles, respectively (Table 1).

**Table 1:** Sociodemographic characteristics of respondents in Ethiopia from March 21 to June 28, 2019 (*N* = 8,663)

Variables	Weighted frequency	Percent	
Residence			
Rural	5,999	69.25	
Urban	2,664	30.75	
Age in year			
15–29	1,670	19.28	
30–39	2,189	25.27	
40–49	1,738	20.06	
50–59	1,236	14.27	
> = 60	1,830	21.13	
Wealth quantile			
Poorest	1,498	17.30	
Poorer	1,634	18.87	
Middle	1,675	19.34	
Richer	1,738	20.06	
Richest	2,117	24.44	
Level of education			
No education, preschool	4,120		47.56
Primary	3,069		35.43
Secondary	872		10.07
Higher	601		6.94
Region			
Tigray	585		6.75
Afar	88		1.01
Amhara	2,110		24.35
Oromia	3,207		37.02
Somali	419		4.83
Benishangul Gumuz	94		1.08
SNMP	1,668		19.25
Gambella	35		0.41
Harari	25		0.29
Addis Ababa	376		4.34
Dire Dawa	57		0.66
Sex			
Male	6,751		77.93
Female	1,912		22.07
Family size			
< = 5	5,516		63.68
> 5	3,147		36.32
Has radio			
Yes	2,411		27.83
No	6,252		72.17
Has television			
Yes	1,458		16.83
No	7,205		83.17

### CBHI coverage by regions in Ethiopia

**Fig. 1:**
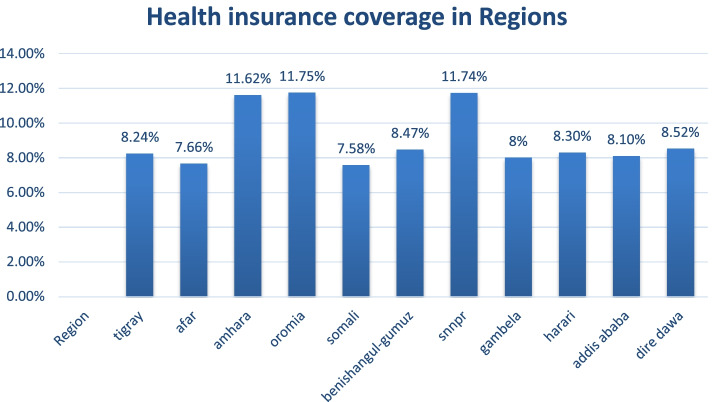
Community based health coverage by regions in Ethiopia, EDHS 2019

In Ethiopia (in 2019), the overall weighted health insurance coverage showed higher coverages in Amhara, SNNPR, whereas lower in regions of Somali, Afar and Gambela with an overall coverage of around 28%. (Fig. 1).

### Spatial distribution of CBHI coverage

**Fig. 2:**
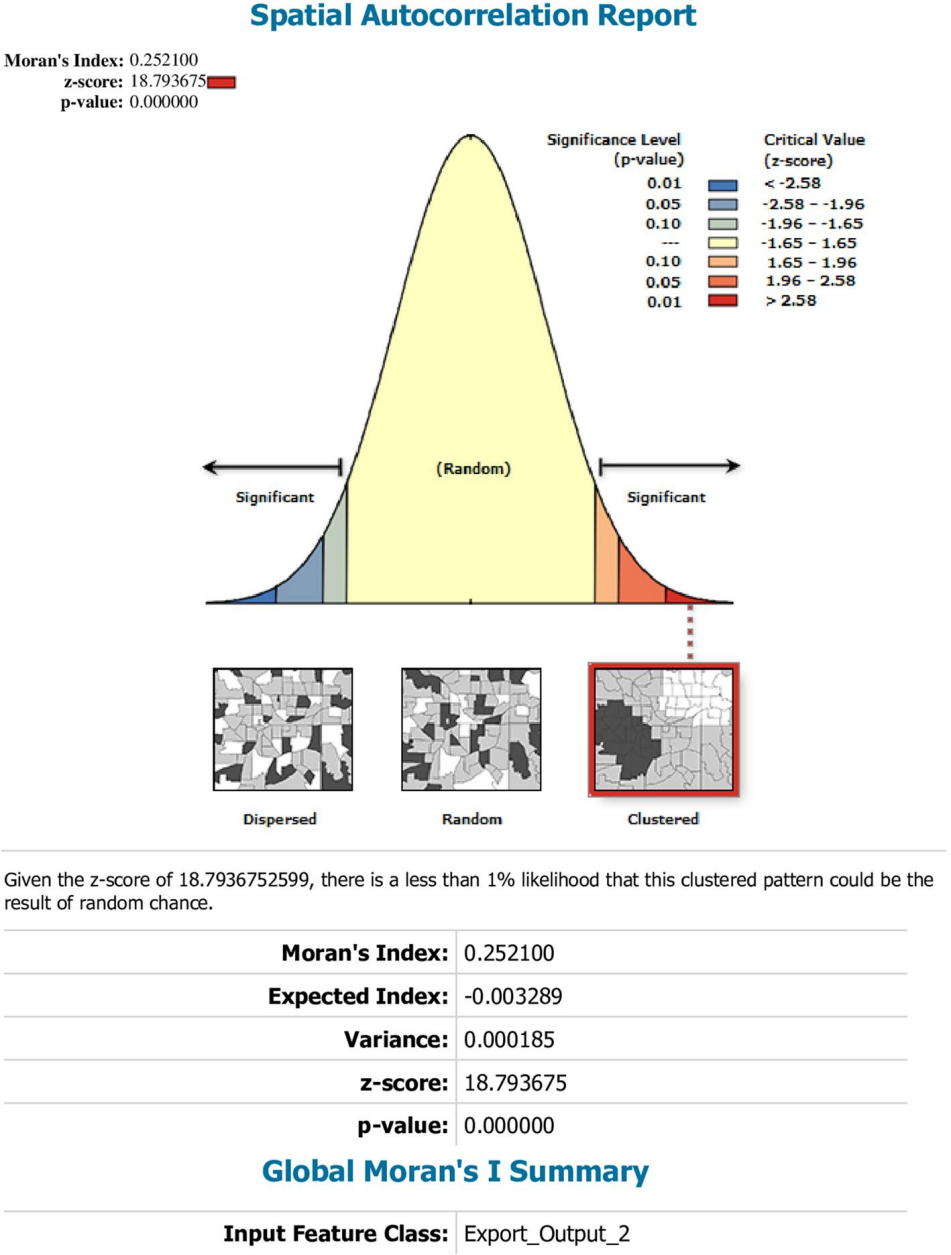
Spatial autocorrelation report analysis of community-based health insurance coverage in Ethiopia, 2019

**Fig. 3:**
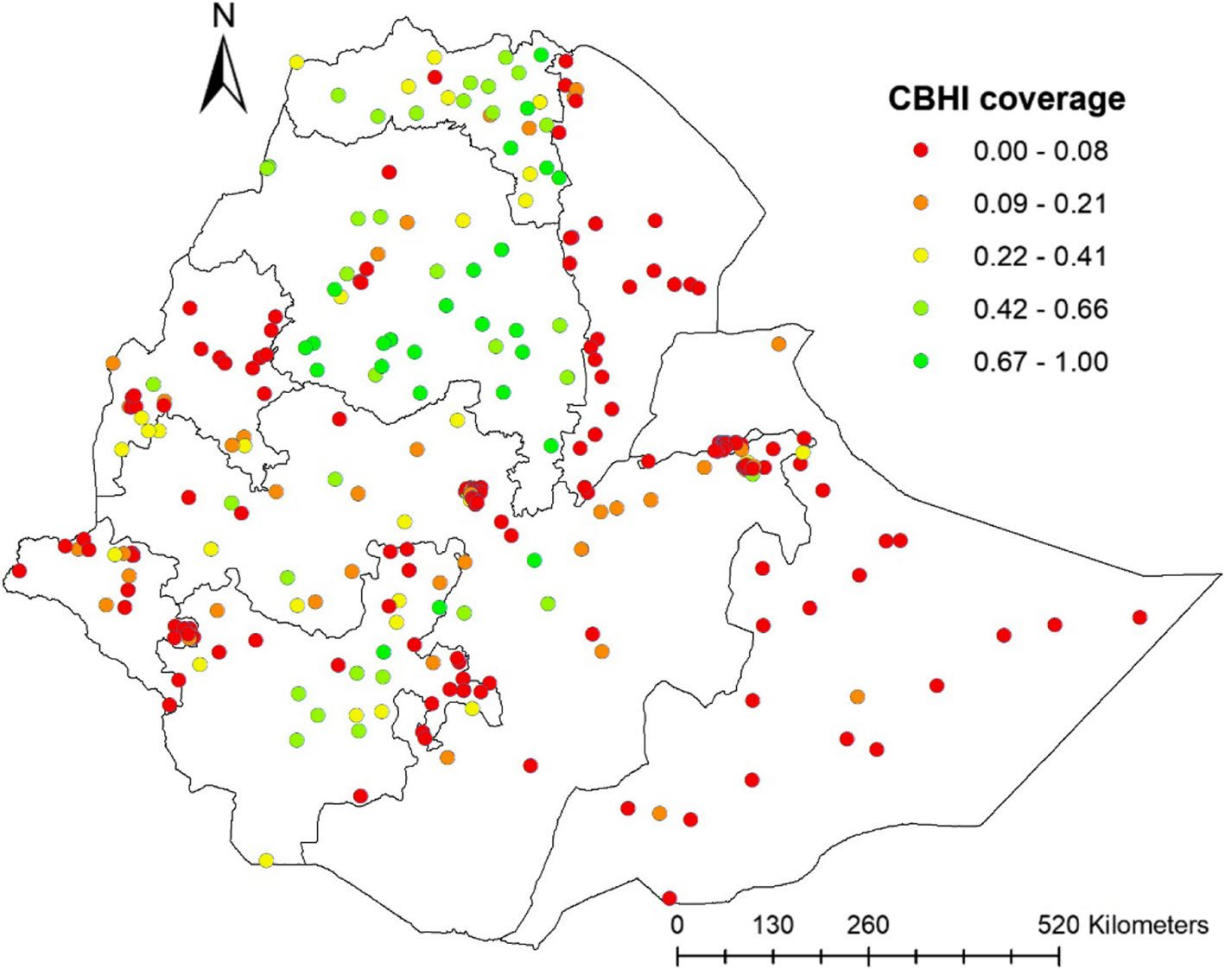
Spatial distribution of a community-based health insurance coverage across regions in Ethiopia, 2019

This study depicted that the spatial distribution of CBHI coverage was spatially clustered in Ethiopia with Global Moran's I 0.252 (*p* < 0.0001). A cluster of high rates in health insurance coverage was observed over the study area. The outputs were automatically generated keys on each panel's right and left sides respectively. Given the z-score of 18.79, there is less than a 1% likelihood that this clustered pattern could result from chance. The bright red and blue colors to the end tails indicates an increased significance level (Fig. 2). Spatial clustering of health insurance coverage was found at regional levels. Of 8,663 households interviewed in 2019, only 2,426 (28.1%) had health insurance coverage. The highest health insurance coverage was spatially clustered in Amhara, Tigray, the central part of SNNPR, some parts of Oromia and Benishangul regions, while Harari, Somali, Afar, Diredawa and Gambella regions had the lowest health insurance coverage (Fig. 3).

### Spatial SaTScan analysis of CBHI Coverage (Bernoulli based model)

Most likely (primary clusters) and secondary clusters of health insurance coverage were identified. In EDHS 2019, spatial scan statistics identified a total of high and modest performing spatial clusters of health insurance coverage. Of these, the spatial scan analysis demonstrated that a total of 81 significant clusters, of which the primary, secondary, tertiary, quaternary, quinary and senary clusters consisted of sixty-five clusters, ten clusters, two clusters, two clusters, one cluster, and one cluster respectively. The primary sat scan window was detected in Amhara, Tigray, a small part of zone two and four in Afar, and some part of Metekel and Agew Awi in Benishangul regions at (12.322718 N, 37.959425 E with a radius value of 265.25 km. This cluster window comprises 1859 population and 874 cases with a relative risk of 3.67 and Log-Likelihood Ratio (LLR) of 463.18 at a p-value of 0.0001. The health insurance coverage was 2.46 times higher to utilize health insurance services than other parts outside the window in this scanning window. The bright blue colours (rings) indicate the most statistically significant spatial windows of health insurance coverage. There was high insurance coverage within the cluster than outside the cluster.

The secondary cluster was also identified by SaT Scan, the most likely cluster and did not overlap spatially with the most likely cluster. The secondary SaTScan circular window is dominantly observed at Ethiopia's SNNPR region (Dawro, Gamo Gofa and Wolayita zones) at (6.272978 N, 36.862733 E) with a distance of 124.08 km. This cluster also has 292 population, and 119 cases with a relative risk of 2.10 and 33.52 are the likelihood of it with a p-value of 0.0001. This cluster was statistically interpreted as health insurance coverage inside the circular window had a 2.10 times higher likelihood of having access to it than households outside the window. Similarly, the SaTscan window analysis demonstrated that the third cluster located predominantly at Central Oromia of southwest Shewa and Arsi zones at (7.531183 N, 38.662596 E) with a radius of 34.42 km. Furthermore, it consists of s57 and 60 cases with a relative risk of 3.54, and the Log-likelihood ratio is 33.45 with the p-value of 0.0001. The study population inside the circular window had a 3.54 times chance of being a user of health insurance service in contrast to the household population outside the spatial window.

**Fig. 4:**
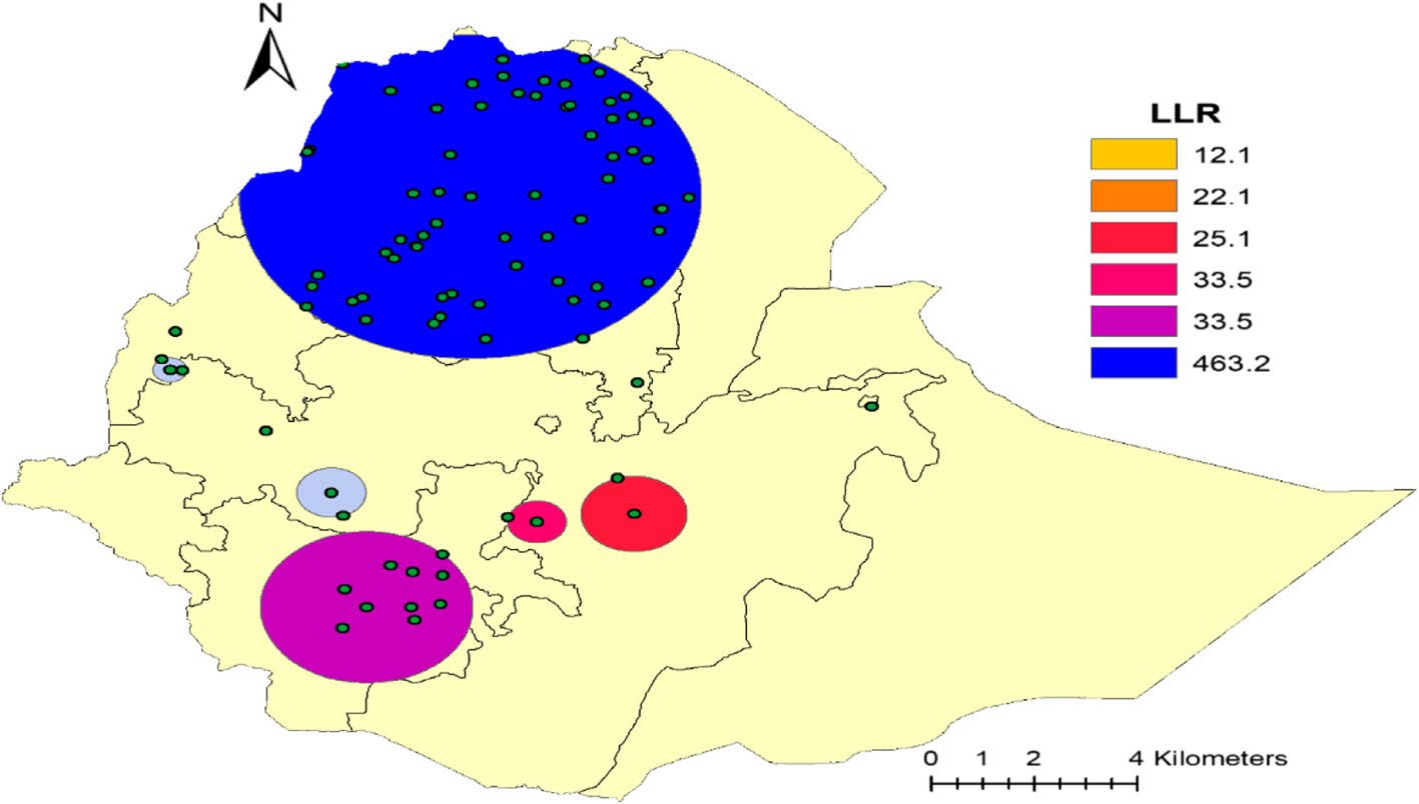
Primary and secondary clusters of health insurance coverage among households across regions in Ethiopia, 2019

The Quaternary cluster is geostatistically located (7.648661 N, 39.688764 E) / and with a radius of 61.78 km and not overlapping spatially with another significant cluster. 57 and 36 populations and cases existed respectively in this cluster with a relative risk of 3.18 and a log-likelihood ratio of 22.14 with a *P*-value of 0.0001. Compared with households outside the circular window, the household inside the circular window had 3.18 times the probability of becoming a health insurance user. The Quinary and Senary clusters also have located geostatistically (9.227458 N, 42.199756 E) / 0 km and (9.227458 N, 42.199756 E) / 0 km and are not overlapping spatially with other significant clusters, respectively. Each of them has 28 and 29 populations; 22 and 18 cases existed respectively in these clusters with a relative risk of 3.94, 3.10 and log-likelihood ratio of 22.14, 12.12 with a *P*-value of 0.0001, 0.0024. Compared with households outside the circular window, those households inside the circular window had a 3.94 and 3.10-times probability of becoming health insurance users, respectively (Table 2 and Fig. 4).

**Table 2:** Significant spatial clusters with high-rate CBHI coverage among household members in Ethiopia, 2019

Cluster	Enumeration areas (Cluster detected)	Coordinates (radius)	population	Cases	RR	LLR	*P*-value
1	83, 82, 57, 84, 56, 78, 58, 59, 54, 81, 74, 61, 60, 62, 75, 53, 9, 22, 18, 70, 76, 23, 65, 20, 71, 7, 13, 2, 8, 14, 21, 72, 55, 24, 85, 1, 5, 51, 79, 63, 12, 19, 52, 11, 165, 80, 46, 29, 17, 44, 6, 25, 73, 162, 77, 66, 36, 3, 64, 45, 16, 10, 163, 4, 67	(12.322718 N, 37.959425 E) / 265.25 km	1859	874	3.67	463.18	0.0001
2	173, 196, 192, 198, 199, 204, 191, 197, 190, 189	(6.272978 N, 36.862733 E) / 124.08 km	292	119	2.1	33.52	0.0001
3	116, 203	(7.531183 N, 38.662596 E) / 34.42 km	57	40	3.54	33.45	0.0001
4	03, 104	(7.648661 N, 39.688764 E) / 61.78 km	57	36	3.18	22.14	0.0001
5	69	(9.577575 N, 39.728117 E) / 0 km	28	22	3.94	22.14	0.0001
6	250	(9.227458 N, 42.199756 E) / 0 km	29	18	3.10	12.12	0.0024
7	97,96	(7.964041 N, 36.490345 E) / 40.33 km	60	25	2.08	7.23	0.139
8	149	(10.333829 N, 34.842459 E) / 0 km	30	15	2.49	6.64	0.241
9	92	(8.880101 N, 35.804185 E) / 0 km	30	13	2.16	4.14	0.877
10	155, 86, 154	(9.766226 N, 34.782318 E) / 19.60 km	90	29	1.61	3.65	0.954

### Spatial interpolation

**Fig. 5:**
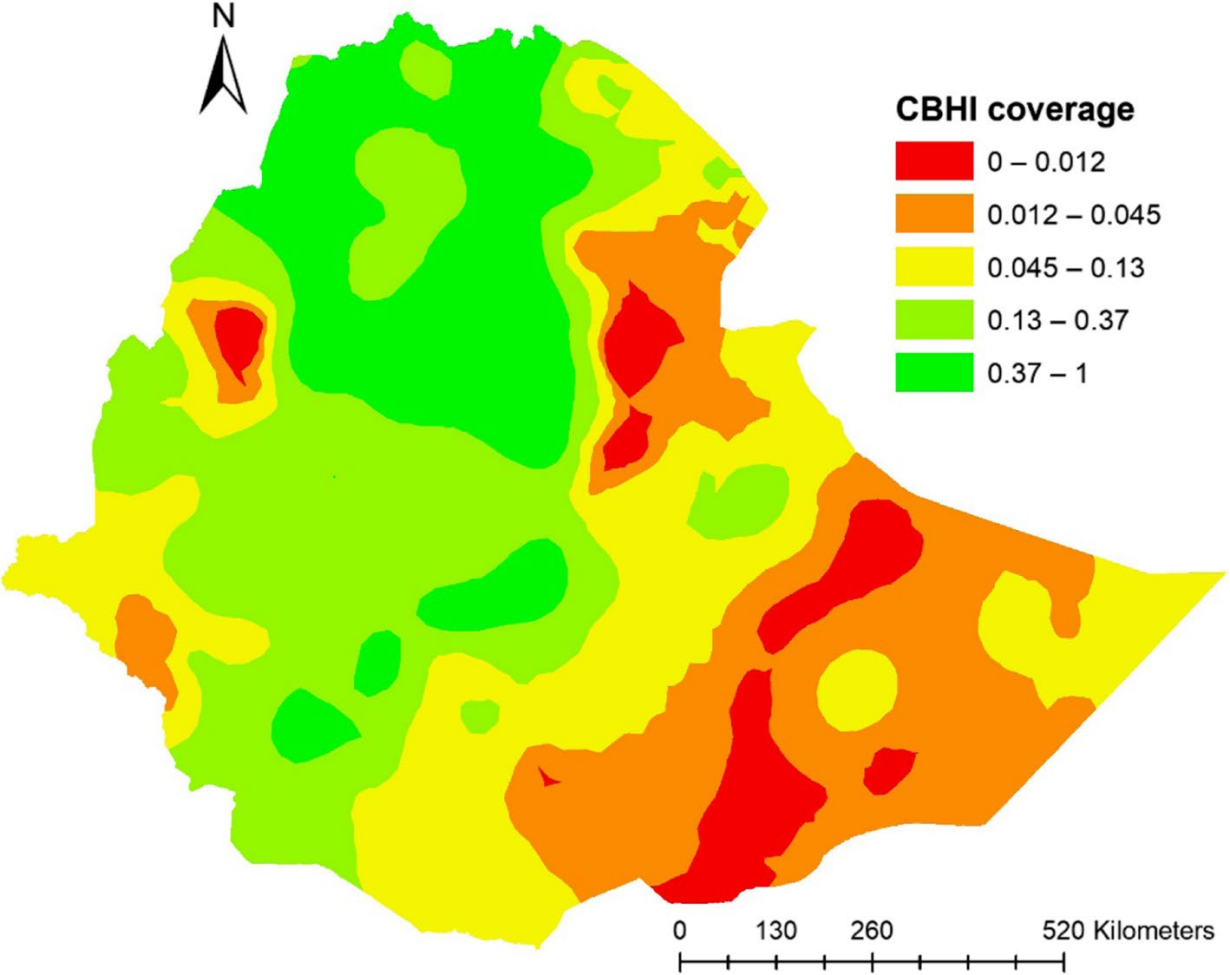
Interpolation predictions of community-based health insurance coverage in Ethiopian across regions, Ethiopia, 2019

Using the Kriging interpolation technique generates high coverage prediction of health insurance, which was identified in some areas of Ethiopia, including almost most parts of Amhara and Tigray regions. The following central Oromia and northeaster of SNNPR regions have also revealed from 13 to 37% of coverage. On the contrary, low coverage prediction was observed in southern and northeastern parts of Somali, south Afar, Gambella and central part of Benishangul Gumuz regions (Fig. 5).

### Individual and community-level factors associated with health insurance in Ethiopia

#### The random effect analysis result

In the null model, the value of ICC was 39.3% which implies the total variability in health insurance was a result of the difference between clusters, whereas the remaining 60.7% was attributable to individual differences. The combined individual and community level model (model III) was the best-fitted model because it had the lowest deviance value in contrast to other models.

#### The fixed effect analysis result

There are various variables run in the multivariable multilevel logistic regression analysis include age, educational attainment, wealth index, sex, having radio and television, family size, place of residence, region. Among these variables, age, education level, wealth index, family size and region were significantly associated with health insurance coverage.

The odds of the age group of 15–29 and 30–39 years being enrolling in health insurance scheme was 0.46(AOR = 0.46, CI: 0.36,0.60) and 0.77(AOR = 0.77, CI: 0.63,0.96) times lower than among age groups of >  = 60 years.

Regarding educational attainments, the probability of Primary educator household members enrolling in a community health insurance scheme was 1.57(AOR = 1.57, CI: 1.15,2.15) times higher than those who did have a higher educational background, respectively..

By their wealth index, middle and richer classes' probability of enrolling in a community health insurance scheme was 1.71(AOR = 1.71, CI: 1.30,2.24) and 1.79(AOR = 1.79, CI: 1.34,2.41) times higher than the poorest class, respectively.

Those households with a member of more than five have a probability of being a member of the community health insurance scheme was 0.82(AOR = 0.82, CI: 0.69,0.96) times lower than households with less than or equal to five children.

The likelihood of experiencing community health insurance by region in Afar, Oromia, Somali, Benishangul Gumuz, SNNPR, Gambella, Harari, Addis Ababa and Dire Dawa was 0.002(AOR = 0.002, CI: 0.006,0.04), 0.11(AOR = 0.11, CI: 0.06,0.21) 0.02(AOR = 0.02, CI: 0.007,0.04), 0.04(AOR = 0.04, CI: 0.02,0.08), 0.09(AOR = 0.09, CI: 0.05,0.18), 0.004(AOR = 0.004, CI: 0.02,0.08), 0.06(AOR = 0.06, CI: 0.03,0.14), 0.07(AOR = 0.07, CI: 0.03,0.16) and 0.03(AOR = 0.03, CI: 0.02,0.07) times less likely utilized than their counterparts Amhara region respectively(Table 3).

**Table 3:** Multivariable multilevel logistic regression analysis of individual and community-level factors associated with health insurance coverage in Ethiopia, EDHS 2019

Individual and community-level variables	Models			
	**Null model** **AOR (95%CI)**	**Model I** **AOR (95%CI)**	**Model II** **AOR (95%CI)**	**Model III** **AOR (95%CI)**
**Age**				
15–29		0.48(.37,0.60)		0.46(0.36,0.60) *
30–39		0.82(0.67,1.01)		0.77(0.63,0.96) *
40–49		1.09(0.88,1.34)		1.01(0.81,1.24)
50–59		1.13(0.90,1.42)		1.08(0.86,1.36)
> = 60		1		1
**Residence type**				
Urban			1	1
Rural			1.96(1.19,3.22)	0.73(0.45,1.19)
**Level of education**				
No education		1.65(1.21,2.34)		1.26(0.89,1.75)
Primary		1.88(1.38,2.56)		1.57(1.15,2.15) *
Secondary		1.45(1.03,2.05)		1.38(0.97,1.95)
Higher		1		1
**Wealth index**				
Poorest		1		1
Poorer		1.32 (1.03,1.70)		1.17(0.91,1.51)
Middle		1.91 (1.46,2.49)		1.71(1.30,2.24) *
richer		1.93 (1.45, 2.55)		1.79(1.34,2.41) *
Richest		1.28 (0.91,1.78)		1.43(0.95,2.17)
**Sex**				
Female		1		1
Male		1.24 (1.05, 1.45)		1.11(0.93,1.32)
**Family size**				
< = 5		1		1
> 5		0.69 (0.59,0.79)		0.82(0.69,0.96) *
**Has radio**				
Yes		1		1
No		0.85 (.72,0.99)		0.92(0.78,1.09)
Has television				
Yes		1		1
No		0.90 (0.70,1.16)		0.93(0.68,1.26)
**Region**				
Tigray			0.67 (.33.1.33)	0.67(0.34,1.33)
Afar			0.01(0.004, 0.28)	0.002(0.006,0.04) *
Amhara			1	1
Oromia			0.13(0.68,0.25)	0.11(0.06,0.21) *
Somali			0.01(0.005,0.03)	0.02(0.007,0.04) *
Benishangul			0.04(0.02,0.09)	0.04(0.02,0.08) *
SNMP			0.11(0.06,0.22)	0.09(0.05,0.18) *
Gambela			0.03(0.02,0.07)	0.004(0.02,0.08) *
Harari			0.06(0.03,0.13)	0.06(0.03,0.14) *
Addis Ababa			0.05(0.03,0.12)	0.07(0.03,0.16) *
Dire Dawa			0.03(0.02,0.06)	0.03(0.02,0.07) *
**Random variables and model comparison**				
PCV (%)	Reference	8.12	59.53	61.1
ICC (%)	54.42	52.32	32.58	31.74
MOR (95% CI)	6.57(5.39,8.21)	6.08(5.01,7.55)	3.31(2.89,3.87)	1.45(2.82,3.78)
Log likelihood (LLR)	-3181.42	3115.30	-3084.13	-3022.51
DIC (2LLR)	6,362.84	6,230.60	6,168.26	6,045.02

*ICC* Intraclass correlation Coefficient, *AOR* Adjusted Odds Ratio, *SNNPR* Southern Nation, Nationalities, & people’s region

^*^Indicates statistically significant variables at *p* value < 0.05

## Discussion

This study assessed and identified the spatial distribution of community health insurance scheme coverage and its associated factors in Ethiopia using the mini-EDHS of 2019 data. The study analyzed that the community health insurance coverage among household members greatly varied across regions in Ethiopia. The Global Moran's I value of 0.252 (*p* < 0.0001) illustrated a significant clustering of community health insurance coverage in the study area.

The highest community health insurance coverage was recorded in Amhara and Tigray regions, and relatively higher reports were observed in the Central part of SNNPR, some parts of Oromia and A nearly high coverage were revealed in Benishangul, a more significant part of SNNPR and Oromia region whereas, lesser health insurance coverage were showed in Somali, Afar, Harari and Dire Dawa regions. All those regions that health insurance coverage recorded lower might be due most of those regions are economically immature and founded at the lowest level of investment in all aspects [34], and in some areas, people are mobile and pastoralist farmers [35]. Another possible factor could be referral and tertiary hospitals number in these regions, which are very small and situated several miles from the home of residents; this might decrease their interest in engaging in health insurance [36].

Regarding Addis Ababa and Dire Dawa regions, since these are the most densely populated cities in the country with many soft options, residents might be chosen to go to private health institutions to receive a high-quality health care service [37] and due to this, they may have less willingness to pay to the CBHI [21]. On the contrary, Amhara and Tigray regions are likely more uncomplicated and faster in methods and systems, and health insurance workers might be more consistent with their activities. In other words, people in these regions may be benefited from any hospitals in the regions. This will enhance access to health insurance [38].

In this study age of the respondent was found as a predictor of community-based health insurance coverage. Relatively speaking, as respondents get older and older, their desire to get community health insurance increases. This finding is in agreement with studies done in Ghana [36, 39, 40], Nigeria [41], Kenya, Nigeria and Tanzania [12], Ethiopia [4, 29]. This may be since as people get older, their desire for health insurance increases because they believe that their immune system is weakening and may also believe that they become more susceptible to illness. In addition to this, older people may become more concerned about their health, and they might not be challenged in shortage of money to pay the health insurance premium because they have more chance to become employed and independent compared to youngers.

Concerning the wealth index, the middle and richer classes have shown a more significant odds ratio to utilize the health insurance coverage than the poorest; however, the poorer and richest classes have lower odds to enroll on the community health insurance. This could not be a financial issue because the total payment amount in Ethiopia is less than $10 per year [42]. Therefore, almost everyone can access health insurance by this amount. Nevertheless, there might be another hidden factor, whether poor awareness about the merit of health insurance among the richest and poorer wealth index groups. This finding is almost similar to studies done, Kenya [43], Namibia [44], Ghana [40], Cameroon [45], and in Ethiopia [4, 29].

Even if it is well-known that individuals with higher levels of education are healthier than individuals with lower levels of education, it is essential to establish whether the relationship between education and health choices is causal or merely represents a correlation in order to assess the total social benefit of education and to determine the potential impact of education policies on health [46]. All though, it is a fact that, when the level of education increases, the perception and knowledge of health insurance benefit will also increase, in the case of educational level, this study found that people in the primary education level are more likely to benefit from health insurance than those with higher education level. This finding is incongruent with a study conducted in Ethiopia [14] and unless people did not have the information and awareness of CBHI, they will not join CBHI because of only they are educated [19]. People in rural areas are more likely to drop out of school. Perhaps the Ethiopian government has created a strong awareness among those uneducated and low-income people to use health insurance coverage in the past years. Government officials have been done without restriction to include all uneducated rural residents; this, in turn, might increase the number of uneducated and primary educated health insurance users [47, 48].

On the other big issue, this study also revealed that households that have more than five members are less likely to join the community health insurance premium chance. It is expected that households with many families' sizes in number will have more chances to join the community-based health insurance scheme due to the substantial financial burden faced by households with a large size when they seek health care services. However, here is the opposite found. Possible justifications might be, these people may not have the proper perspective attitude and knowledge on community-based health insurance coverage. On the contrary, they may be negligent and view early premium payment as a crisis. Another possible idea might be that these people may think they are entirely healthy and less likely to seek medical attention because they may not be aware of the symptoms and signs of any health-related issues. Furthermore, reasons might arise if there are bureaucratic obstacles, low reimbursement rates, poor service quality, and a long time of waiting for treatment and other barriers for members to use health insurance, especially for those new to the systems [49].

## Conclusion

This study concludes that community-based health insurance coverage among households in Ethiopia is deficient still. Additionally, sociodemographic factors such as age, education, geopolitical regions, and family size were significant predictors of enrolment in community-based health insurance in Ethiopia. This study found the highest rate of health insurance coverage in the Amhara and Tigray regions, and also it identified a lower cluster of health insurance coverage rates in regions such as Afar, Somali, Harari, Dire Dawa, and Benishangul. There is an incompatibility of community health insurance coverage in Ethiopia across regions; hence, the Ethiopian government needs to develop consistent support financially and technically and create awareness to those regions with lower health insurance coverage.

## Data Availability

All necessary information's were included within the manuscript.

## Abbreviations

AOR: Adjusted Odds Ratio
CBHI: Community Based Health Insurance
CSA: Central Statistics Agency;
DIC: Deviance Information Criteria
EA: Enumeration Area
EDHS: Ethiopian Demographic and Health Survey
GLMM: Generalized Linear Mixed Model
HSDP: Health Sector Development Plan
ICC: Intraclass Coefficient
LLR: Log Likelihood Ratio
SNNPR: Southern Nation and Nationality and Peoples Region
VIF: Variance Inflation Factor
WHO: World Health Organization

## References

[1] Donfouet HPP, Mahieu P-A (2012). Community-based health insurance and social capital: a review. Heal Econ Rev.

[2] Saksena P, Hsu J, Evans DB (2014). Financial risk protection and universal health coverage: evidence and measurement challenges. PLoS Med.

[3] Mathauer I, Torres LV, Kutzin J, Jakab M, Hanson K (2020). Pooling financial resources for universal health coverage: options for reform. Bull World Health Organ.

[4] Kebede SA, Liyew AM, Tesema GA, Agegnehu CD, Teshale AB, Alem AZ (2020). Spatial distribution and associated factors of health insurance coverage in Ethiopia: further analysis of Ethiopia demographic and health survey, 2016. Arch Public Health.

[5] Demographic E (2012). Health Survey 2011 Addis Ababa, Ethiopia and Calverton, Maryland, USA: Central Statistical Agency and ICF International.

[6] Mebratie AD, Sparrow R, Yilma Z, Abebaw D, Alemu G, Bedi A (2013). Impact of Ethiopian pilot community-based health insurance scheme on health-care utilisation: a household panel data analysis. Lancet.

[7] Shewamene Z, Tiruneh G, Abraha A, Reshad A, Terefe MM, Shimels T (2021). Barriers to uptake of community-based health insurance in sub-Saharan Africa: a systematic review. Health Policy Plan.

[8] Bump J, Cashin C, Chalkidou K, Evans D, González-Pier E, Guo Y (2016). Implementing pro-poor universal health coverage. Lancet Glob Health.

[9] Maeda A, Araujo E, Cashin C, Harris J, Ikegami N, Reich MR. Universal Health Coverage for Inclusive and Sustainable Development: A Synthesis of 11 Country Case Studies. Directions in Development--Human Development. Washington, DC: World Bank. © World Bank; 2014. https://openknowledge.worldbank.org/handle/10986/18867. License: CC BY 3.0 IGO. URI http://hdl.handle.net/10986/18867.

[10] Etienne C, Asamoa-Baah A, Evans DB. Health systems financing: the path to universal coverage. Geneva: World Health Organization; 2010. 10.2471/BLT.10.078741 PMC2878164 20539847

[11] Evans DB, Hsu J, Boerma T. Universal health coverage and universal access. Geneva: World Health Organization; 2013. 10.2471/BLT.13.125450. PMC3738317 23940398

[12] Amu H, Dickson KS, Kumi-Kyereme A, Darteh EKM (2018). Understanding variations in health insurance coverage in Ghana, Kenya, Nigeria, and Tanzania: evidence from demographic and health surveys. PLoS ONE.

[13] Tilahun H, Atnafu DD, Asrade G, Minyihun A, Alemu YM (2018). Factors for healthcare utilization and effect of mutual health insurance on healthcare utilization in rural communities of South Achefer Woreda, North West. Ethiopia Health Econ Rev.

[14] Gidey MT, Gebretekle GB, Hogan M-E, Fenta TG (2019). Willingness to pay for social health insurance and its determinants among public servants in Mekelle City, Northern Ethiopia: a mixed methods study. Cost Eff Resour Alloc.

[15] Mebratie AD, Sparrow R, Yilma Z, Alemu G, Bedi AS (2015). Dropping out of Ethiopia’s community-based health insurance scheme. Health Policy Plan.

[16] Feleke S, Mitiku W, Zelelew H, Ashagari T (2015). Ethiopia’s Community-based Health Insurance: A step on the road to universal health coverage. Case Study for the USAID.

[17] Csa I (2016). Ethiopia Demographic and Health Survey; Central Statistical Agency Addis Ababa, Ethiopia.

[18] Agency EHI (2015). Evaluation of community‐based health insurance pilot schemes in Ethiopia.

[19] Agago TA, Woldie M, Ololo S (2014). Willingness to join and pay for the newly proposed social health insurance among teachers in Wolaita Sodo town, South Ethiopia. Ethiop J Health Sci.

[20] Belaynesh Abebaw. ea. “Willingness to Pay for the Newly Proposed Social Health Insurance Scheme and Associated Factors Among Civil Servants in Debre Markos Town, North West Ethiopia. Med Res Clin Case Rep. 2015;22 (2018):164–177.

[21] Lasebew Y, Mamuye Y, Abdelmenan S (2017). Willingness to pay for the newly proposed social health insurance among health workers at St. Paul’s Hospital Millennium Medical College, Addis Ababa, Ethiopia. Int J Health Econ Policy..

[22] Bantie GM, Woya AA, Zewdie BM (2020). Community-Based Health Insurance and Associated Factors in North-Western Ethiopia. The Case of Bahir Dar City. Int J Gen Med.

[23] Fite MB, Roba KT, Merga BT, Tefera BN, Beha GA, Gurmessa TT (2021). Factors associated with enrollment for community-based health insurance scheme in Western Ethiopia: Case-control study. PLoS ONE.

[24] Health FDRoEMo. Health Sector Development Program IV: 2010/11–2014/15. Ministry of Health Addis Ababa: Federal Democratic Republic of Ethiopia Ministry of Health; 2010. Available from: https://www.healthynewbornnetwork.org/hnn-content/uploads/HSDP-IV-Final-Draft-October-2010-2.pdf.

[25] CSA I (2017). Central Statistical Agency (CSA)[Ethiopia] and ICF. Ethiopia Demographic and Health Survey 2016.

[26] Factbook CW. Ethiopian Demographics Profile. Index Mundi; 2021. Available from: https://www.indexmundi.com/ethiopia/demographics_profile.html.

[27] Ethiopian Public Health Institute - EPHI, Federal Ministry of Health - FMoH, ICF (2019). Ethiopia Mini Demographic and Health Survey 2019.

[28] Ephiee I (2019). Ethiopia mini demographic and health survey 2019: key indicators.

[29] Atnafu DD, Tilahun H, Alemu YM (2018). Community-based health insurance and healthcare service utilisation, North-West, Ethiopia: a comparative, cross-sectional study. BMJ Open.

[30] Koo TK, Li MY (2016). A guideline of selecting and reporting intraclass correlation coefficients for reliability research. J Chiropr Med.

[31] Portney LG, Watkins MP (2009). Foundations of clinical research: applications to practice: Pearson/Prentice Hall Upper Saddle River, NJ.

[32] Spiegelhalter DJ, Best NG, Carlin BP, Van Der Linde A (2002). Bayesian measures of model complexity and fit. Journal of the royal statistical society: Series b (statistical methodology).

[33] Kulldorff M. SaTScan (TM) User Guide for version 7.0. SaTScanTM Accessed on August. 2006;13:2007. Available from http://cdemat.free.fr/iurc/demattei/satscan.7.0/SaTScan_Users_Guide.pdf.

[34] Seid M, Lamesegen T (2019). Regional Disparity Of Investment In Ethiopia From 1992–2017. Noble Int J Soc Sci Res.

[35] Ethiopia U (2019). Education for Pastoralist Children.

[36] Manortey S, VanDerslice J, Alder S, Henry KA, Crookston B, Dickerson T (2014). Spatial analysis of factors associated with household subscription to the National Health Insurance Scheme in rural Ghana. J Public Health Afr.

[37] Abdilwohab MG, Abebo ZH, Godana W, Ajema D, Yihune M, Hassen H (2021). Factors affecting enrollment status of households for community based health insurance in a resource-limited peripheral area in Southern Ethiopia. Mixed method. Plos one.

[38] Yilma Z, Mebratie A, Sparrow R, Dekker M, Alemu G, Bedi AS (2015). Impact of Ethiopia's community based health insurance on household economic welfare. World Bank Econ Rev..

[39] Badu E, Agyei-Baffour P, Ofori Acheampong I, Preprah Opoku M, Addai-Donkor K. Households sociodemographic profile as predictors of health insurance uptake and service utilization: a cross-sectional study in a municipality of Ghana. Advances Public Health. 2018;2018:7–10.

[40] Duku SKO (2018). Differences in the determinants of health insurance enrolment among working-age adults in two regions in Ghana. BMC Health Serv Res.

[41] Aregbeshola BS, Khan SM (2018). Predictors of enrolment in the National Health Insurance Scheme among women of reproductive age in Nigeria. Int J Health Policy Manag.

[42] Agency EHi (2015). Evaluation of Community-Based Health Insurance Pilot Schemes in Ethiopia: Final Report.

[43] Kimani JK, Ettarh R, Warren C, Bellows B (2014). Determinants of health insurance ownership among women in Kenya: evidence from the 2008–09 Kenya demographic and health survey. Int J Equity Health.

[44] Allcock SH, Young EH, Sandhu MS (2019). Sociodemographic patterns of health insurance coverage in Namibia. Int J Equity Health.

[45] Donfouet HPP, Makaudze E, Malin E, Edimo J-RE. The economic value of the willingness-to-pay for a community-based prepayment scheme in rural Cameroon. Research Paper. 2011;3:2, 11. 10.1007/s10754-011-9097-3 21874541

[46] Raghupathi V, Raghupathi W (2020). The influence of education on health: an empirical assessment of OECD countries for the period 1995–2015. Arch Public Health.

[47] Mebratie AD, Sparrow R, Yilma Z, Alemu G, Bedi AS (2015). Enrollment in Ethiopia’s community-based health insurance scheme. World Dev.

[48] Shigute Z, Strupat C, Burchi F, Alemu G, Bedi AS (2017). The joint effects of a health insurance and a public works scheme in rural Ethiopia.

[49] Liu X, Tang S, Yu B, Phuong NK, Yan F, Thien DD (2012). Can rural health insurance improve equity in health care utilization? A comparison between China and Vietnam. Int J Equity Health.

